# Familial Screening for Left-Sided Congenital Heart Disease: What Is the Evidence? What Is the Cost?

**DOI:** 10.3390/diseases5040029

**Published:** 2017-12-08

**Authors:** Daniel J. Perry, Connor R. Mullen, Horacio G. Carvajal, Anoop K. Brar, Pirooz Eghtesady

**Affiliations:** Division of Pediatric Cardiothoracic Surgery, Washington University School of Medicine, St. Louis, MO 63110, USA; perryd2@mail.uc.edu (D.J.P.); crmqk6@health.missouri.edu (C.R.M.); carvajalhor2@gmail.com (H.G.C.); brara@wustl.edu (A.K.B.)

**Keywords:** familial screening, congenital heart disease, hypoplastic left heart disease, cost-effectiveness, bicuspid aortic valve, familial aggregation

## Abstract

Since the American Heart Association’s recommendation for familial screening of adults with congenital heart disease for bicuspid aortic valve, similar recommendations for other left-sided heart defects, such as hypoplastic left heart syndrome (HLHS), have been proposed. However, defining at-risk populations for these heart defects based on genetics is less straightforward due to the wide variability of inheritance patterns and non-genetic influences such as environmental and lifestyle factors. We discuss whether there is sufficient evidence to standardize echocardiographic screening for first-degree relatives of children diagnosed with HLHS. Due to variations in the inclusion of cardiac anomalies linked to HLHS and the identification of asymptomatic individuals with cardiac malformations, published studies are open to interpretation. We conclude that familial aggregation of obstructive left-sided congenital heart lesions in families with history of HLHS is not supported and recommend that additional screening should adopt a more conservative definition of what truly constitutes this heart defect. More thorough consideration is needed before embracing familial screening recommendations of families of patients with HLHS, since this could inflict serious costs on healthcare infrastructure and further burden affected families both emotionally and financially.

## 1. Introduction

Screening protocols for at-risk populations improve the outcomes of patients with diseases that are asymptomatic by detecting them before they progress far enough to be imminently life-threatening and facilitating their treatment. The challenge in balancing this potentially life-saving tool with cost-effectiveness, while avoiding frequent misdiagnosis, lies in correctly identifying such populations. Likewise, the heritability and associated pathologies of each disease must be taken into consideration when making screening recommendations. In 2008, the American Heart Association recommended familial screening for bicuspid aortic valve (BAV) in their clinical practice guidelines for adults with congenital heart disease (CHD) [1]. Since then, several studies have made similar recommendations for other left-sided heart defects, in particular, hypoplastic left heart syndrome (HLHS) and coarctation of the aorta (CoA) [2,3,4,5]. Understanding the existing data for familial aggregation of these diseases using the tools available for evaluating the efficacy of a screening recommendation is important to determine whether familial screening should be extended to other left-sided heart lesions.

## 2. Methods

A literature review was performed to determine the methodology used in investigating familial clustering of cardiac diseases. We focused on studies concerning cardiac defects because the cost and limitations associated with image-based screening are not necessarily the same for familial clustering/screening protocols for other diseases. The population-based studies used the following basic principles—recruit a cohort of patients with the disease of interest (index cases), construct a pedigree based on family history of first- and second-degree relatives, and lastly, screen first-degree relatives for undiagnosed defects similar to the disease of interest. Studies varied in strength depending on alterations in their methodology, such as including a blind reviewer for validation of image-based diagnoses [6,7].

## 3. Results

### 3.1. Bicuspid Aortic Valve Studies

Familial aggregation of BAV was first noticed in 1977 by Gale et al. [8]. Later, Huntington et al. recruited 210 first-degree relatives of 30 patients with BAV to see if the isolated reports of families with multiple cases of BAV held true in a larger population [9]. Of the 186 subjects they screened by echocardiography, 17 cases of BAV were found. To strengthen the case for utilizing a familial BAV screening protocol, studies have analyzed the heritability of BAV in family populations and analyzed the cost-effectiveness of such screening. To determine the heritability of BAV compared to various cardiovascular malformations (CVM), Cripe et al. screened first-degree relatives of 50 patients with BAV [4]. Using “maximum-likelihood-based variance decomposition extended to dichotomous traits”, they estimated the heritability of BAV in their sample to be 89% and concluded that the suggestion for screening first-degree relatives of those with BAV was warranted [4]. McBride et al., who examined 124 families (351 individuals) with an index case of left ventricular outflow tract malformations, identified CVM in approximately 9% of relatives, 5% of which were BAV cases [10]. In their study, the relative risk for first degree relatives was calculated to be 36.9 with a heritability of 0.71–0.90, prompting them to recommended screening of asymptomatic relatives. Similarly, a large study by Lewin et al. screened 282 asymptomatic first-degree relatives of children with congenital aortic valve stenosis, HLHS, and CoA; their findings led them to recommend echocardiographic evaluation for asymptomatic parents and siblings of individuals with left ventricular outflow tract lesions for cardiovascular anomalies, particularly BAV [3]. 

The cost-effectiveness of screening siblings of BAV patients has been evaluated by Hales et al. [11]. In order to estimate the cost of identifying and preventing an aortic dissection in a sibling of a patient with BAV (based on the assumption that the newly identified subject would get a limited echocardiography every two years), the authors considered: the cost of undergoing an echocardiogram, the probability of detecting a cardiovascular malformation, the probability of experiencing an aortic dissection, life expectancy, and mortality from dissection. They deemed their estimate of $363,911/per individual acceptable, especially when compared to other screening protocols, such as universal echocardiographic screening for CHD [12] or 2D echocardiographic screening of young adult athletes [13]. Collectively, these studies indicating the aggregation and heritability of BAV, as well as the cost-effectiveness of screening first-degree relatives, were overwhelming enough to prompt the American Heart Association and the American College of Cardiology [14] to recommend that all physicians adopt the practice [1]. As in the realm of device and drug approval, however, it would be prudent to test this recommendation in the real world, where the selection of the index case may pose certain challenges. A clear example is the difficulty of diagnosing BAV in a newborn; such ascertainments, if biased, could impact frequency counts, as described in the Cripe study [4].

### 3.2. Screening for Hypoplastic Left Heart Syndrome

Hypoplastic left heart syndrome is another left-sided heart defect with complex genetic influences [15,16,17,18]. Some evidence suggests that first-degree relatives of patients with HLHS have a higher incidence of BAV (12%) [7] compared with the general population (1–2%) [19]. This has raised the question of whether HLHS is another CVM for which familial screening should be implemented, similar to BAV, due to the significant morbidity and mortality associated with HLHS [20,21,22,23]. Studies investigating the heritability of HLHS typically recruit HLHS probands and their first- and second-degree relatives to participate in screening studies with the goal of determining the familial clustering and heritability of this CHD [3,6,15]. Cascade screening is used to systematically identify close relatives of index cases to determine if the relatives may be carriers or unknowingly be affected with a particular disease. In recent years, cascade screening based on genetic testing has been implemented for several diseases, including familial hypercholesterolemia [24,25] and fragile X syndrome [26], for which it is highly beneficial and cost-effective. Amongst CHD, based on current diagnostic strategies, our study suggests that while cascade screening may be an attractive option in the case of BAV, HLHS does not meet the criteria for this strategy given its complicated inheritance. HLHS does not follow simple Mendelian genetics, but rather exhibits “complex inheritance”, with contributions from both genetic [18,27,28] and environmental risk factors [6,29,30]. Overall, while the results of these studies support the notion of familial clustering of HLHS, the evidence is not as overwhelming as for BAV. 

One of the earliest published population screening studies focusing on HLHS was conducted in 1989 by Brenner et al. on data collected in the Baltimore-Washington Infant Study [7]. These investigators recruited first-degree relatives of 14 patients diagnosed with HLHS as defined by “…aortic atresia and mitral stenosis-atresia with hypoplasia of the left ventricle”. Of the 41 first-degree relatives studied, 5 (12%) had a left ventricular outflow tract abnormality. The report concluded that the identification of the affected first-degree relatives could be a coincidence due to a selection bias resulting from the selection of probands with severe rather than mild left-sided heart defects. Alternatively, the CVM identified in the relatives could be caused by a defect in a common gene or set of genes. Regardless of the case, they reasoned that the sample size of their cohort was insufficient to reach a definitive conclusion regarding familial clustering of HLHS. 

Subsequently, a study published eight years later, titled “Hypoplastic Left Heart Syndrome is Heritable”, recruited 38 patients with HLHS and 193 of their family members (126 first-degree relatives) and screened them for CVM [15]. They identified CVM in 24% of the relatives, including HLHS, BAV, CoA, ventricular septal defects (VSD), aortic root dilatation, and left superior vena cava. The incidence of CVM in the first-degree relatives was 23 of 126 (18.3%); 12 of the 126 had isolated BAV (9.5%); one of the 126 had CoA (0.8%); and four out of the 126 (3.2%) had HLHS, two of whom were siblings from the same family (i.e., each proband counted as a first-degree relative for the other). Using a liberal normal population incidence estimate of 0.08%, they determined the heritability to be as high as 0.99, and noted that the pattern of inheritance was likely complex.

### 3.3. Cardiovascular Malformations Associated with HLHS

Evidence of heritability can vary across studies based on which CVM are included in the analyses. For instance, a study by Kelle et al. [2] was much less stringent in the inclusion of what constituted a CVM related to HLHS, and unlike the studies that support BAV familial screening, it did not show a high incidence of a one-to-one association between a disease in a patient and that same disease in relatives. The etiology of natural cardiac anatomical variants, such as patent ductus arteriosus (PDA), anomalous aortic origin of coronary arteries, or patent foramen ovale (PFO), does not involve a pathological error in cardiac formation, but rather may represent a spectrum of normal developmental variations. Moreover, the prevalence and incidence of these and other malformations in the general population need to be considered to contextualize such conclusions. Among these, PFO is found in 15–25% of healthy adults, while persistence of congenital anomalies such as left-sided superior vena cava (considered the most common central venous anomaly) has an incidence of 0.1–0.3% in the general population and 2.1–5% in patients with CHD [31,32,33]. The inclusion of diagnoses of left superior vena cava anomalies, PFO, and aortic root dilatation alongside cardiac defects such as VSD, tetralogy of Fallot, and CoA may be inappropriate and lead to incorrect conclusions and erroneous heritability calculations. Table 1 shows the expected numbers, for example, in a study with a small sample size, as reported by Kelle et al. [2], of how many common cardiovascular anomalies one would diagnose in the general population simply by chance. Inclusion of these common CVM would, therefore, lead to a measurement bias in a small sample size, especially if the pathophysiological mechanism is unclear.

Similarly, partial anomalous pulmonary venous return (PAPVR) is a common CVM reported at high frequency among the general population (Table 1). Ho et al. retrospectively identified 49 asymptomatic adult patients with incidental PAPVR findings from 45,538 contrast-enhanced computed tomography examinations of the chest performed over an eight-year period, reporting a prevalence of at least 0.1% among the general population [38]. Likewise, Hughes et al. reported an incidence of 0.7% of anomalous pulmonary veins in 208 cadavers examined [39]. Another common CVM included in some studies is mitral valve prolapse (MVP) with mitral regurgitation. The prevalence of MVP was reported as 2.4% in adults in the Framingham Heart Study among 3491 subjects [40], and 0.7% in a population of 2072 healthy teenagers [41]. The presence of MVP in the aforementioned study populations clearly shows that this CVM occurs at a significant rate in the general population, and could therefore be expected to occur by chance among a small study population. Thus, without a clear pathophysiological relationship, considering MVP as a CVM finding when screening first-degree relatives of individuals with HLHS could lead to measurement bias.

Three recent studies that have made family screening recommendations for HLHS vary in their definition of CVM linked to HLHS. In contrast to the studies supporting BAV screening, which were based on a one-to-one association of BAV and family members, definitions of HLHS are linked to four or more other CVMs. In the first study, Kelle et al. analyzed the prevalence of CVMs among 152 first-degree relatives of 52 patients diagnosed with HLHS [2]. Based on an 11% incidence of CVM in the study population, the authors suggest that echocardiographic screening should be standard for first-degree relatives of children diagnosed with HLHS. The CVMs included in the list of diagnoses were anomalous aortic origin of the right coronary artery, BAV, bicuspid pulmonary valve, CoA, dilated ascending aorta with normal aortic valve, dilated aortic root with accessory mitral chord tissue, mitral valve prolapse with mitral regurgitation, PAPVR, papillary fibroelastoma, PDA, and VSD. A second study by Loffredo et al. reported a 19.3% incidence of CVM in a population of 135 first-degree relatives of patients diagnosed with HLHS [6]. Although this study limited HLHS-related CVM to atrial septal defects (ASD), BAV, PDA, VSD, and tetralogy of Fallot, it did not clearly state whether they were certain that some of the ASD were not PFO diagnoses. Moreover, this study did not associate an age, gender, or history of intervention with the first-degree relatives diagnosed with ASD. As a result, no inferences can be made about the size of the defects, which could potentially allow one to differentiate an ASD from a PFO. In a third study by Brenner et al., the anomalies considered as CVMs related to HLHS were BAV and mild aortic stenosis, with an incidence of 12.2% in a population of 41 relatives [7]. 

Furthermore, conditions such as aortic root dilatation are associated with a number of disease mechanisms including hemodynamic and blood pressure imbalances, as reported in cohorts of the Strong Heart Study [42] and the Framingham Heart Study [43]. The latter analysis was a longitudinal study over a period of 16 years which demonstrated that aortic root remodeling in mid to late adulthood is common using serial echocardiograms. Their results show that a decade increase in age was associated with a larger aortic root and cardiac remodeling [44]. In the Coronary Artery Risk Development in Young Adults Study, aortic root diameter in healthy young adults (ages 18–30 years) studied using echocardiography over a 20-year time period was found to be modified by smoking, hypertension, and body weight gain [45]. These studies show that environmental and lifestyle factors which can influence the course of certain CVM need to be recognized.

### 3.4. The Cost of Screening

The data regarding familial aggregation of left-sided heart defects besides BAV does not convincingly indicate whether familial screening for defects such as HLHS or CoA should or should not be instituted. As discussed above, population screening studies, as well as cascade familial screening [24,26] can identify asymptomatic defects; however, if improperly implemented, the costs may outweigh any benefits. Based on the average cost of an echocardiogram (approximately $700), if 10–15% of first-degree relatives have a ‘CVM’ lesion (i.e., one would have to perform an echocardiogram evaluation of 10–15 individuals to find one CVM), the cost of identifying the CVM would be approximately $7000–$11,000 per year for that single relative. Using the prevalence of HLHS, 1 in 4344 births [46], and assuming four million births per year in the United States, there are approximately 800 anticipated HLHS births per year. If each one of these probands led to familial screening, it would cost approximately $5.6 million to identify a malformation in 800 relatives (assuming screening of 10 relatives). The question is whether this is a reasonable cost for identifying common congenital anomalies that are asymptomatic or associated with other CVM—such as left superior vena cava [47], PFO, or PDA [48]—among the general population? If the goal is to identify serious CVM with potential life-long consequences, then studies examining the efficacy of familial screening should be carried out with rigorous inclusion criteria.

## 4. Conclusions

In order to prevent unnecessary financial and emotional burden, a more thorough consideration and investigation into the causal mechanisms of CHD is needed before embracing the screening recommendations proposed for families of patients with select CHD such as HLHS. Continued pragmatic trials are necessary to evaluate recommendations made based on small single-center studies.

## Figures and Tables

**Table 1:** Prevalence of common cardiovascular abnormalities in the general population and the expected number of CVM diagnosed by chance in a study with a small sample size. Abbreviations: ASD = atrial septal defect; PAPVR = partial anomalous pulmonary venous return; PDA = patent ductus arteriosus; PFO = patent foramen ovale; PLSVC = persistent left superior vena cava.

Cardiovascular Malformation	Estimated Prevalence in General Population	Expected Number (*N* = 152) Kelle et al. [2]
PFO	148/5581 (25.6%) [34]	40 (26.31%)
PDA	35/56,109 (0.06%) [35]	1 (0.66%)
ASD	523/398,140 (0.13%) [36]	1 (0.66%)
PLSVC	5/5000 (0.1%) [37]	1 (0.66%)
PAPVR	47/45,538 (0.1%) [38]	1 (0.66%)
